# Tuning heat transport via coherent structure manipulation: recent advances in thermal turbulence

**DOI:** 10.1093/nsr/nwad012

**Published:** 2023-01-12

**Authors:** Ke-Qing Xia, Shi-Di Huang, Yi-Chao Xie, Lu Zhang

**Affiliations:** Center for Complex Flows and Soft Matter Research and Department of Mechanics and Aerospace Engineering, Southern University of Science and Technology, Shenzhen 518055, China; Center for Complex Flows and Soft Matter Research and Department of Mechanics and Aerospace Engineering, Southern University of Science and Technology, Shenzhen 518055, China; State Key Laboratory for Strength and Vibration of Mechanical Structures and School of Aerospace, Xi’an Jiaotong University, Xi’an 710049, China; Center for Complex Flows and Soft Matter Research and Department of Mechanics and Aerospace Engineering, Southern University of Science and Technology, Shenzhen 518055, China

**Keywords:** coherent structure, turbulent flow, heat transport, geometrical confinement, dynamical constraint

## Abstract

Tuning transport properties through the manipulation of elementary structures has achieved great success in many areas, such as condensed matter physics. However, the ability to manipulate coherent structures in turbulent flows is much less explored. This article reviews a recently discovered mechanism of tuning turbulent heat transport via coherent structure manipulation. We first show how this mechanism can be realized by applying simple geometrical confinement to a classical thermally driven turbulence, which leads to the condensation of elementary coherent structures and significant heat-transport enhancement, despite the resultant slower flow. Some potential applications of this new paradigm in passive heat management are also discussed. We then explain how the heat transport behaviors in seemingly different turbulence systems can be understood by this unified framework of coherent structure manipulation. Several future directions in this research area are also outlined.

## Teaser

This article reviews a new paradigm on how heat transport in thermal turbulence can be controlled via coherent structure manipulation, which is expected to be generalized in other turbulence systems.

## INTRODUCTION

As the last unsolved problem in classical physics, fluid turbulence is not only of fundamental importance, but also plays a crucial role in a broad range of industrial processes [1]. One of the most prominent features of turbulent flows is the existence of coherent structures [2]. These flow structures are distinct from the random motions of the turbulent background and are more efficient in the global transport of mass, momentum and heat. Therefore, it is very tempting to manipulate these elementary structures to control turbulent transport, which has huge applications in numerous engineering processes. However, strong dissipations are inevitable and intrinsic in turbulent flows, so it is notoriously difficult to manipulate coherent structures in these systems. This is particularly the case for thermal turbulence, as heat flows and thermal structures are inherently associated with random motions and dissipations. Because of these reasons, studies on the manipulation of coherent structures to control turbulent transport remain scarce.

**Figure 1.**
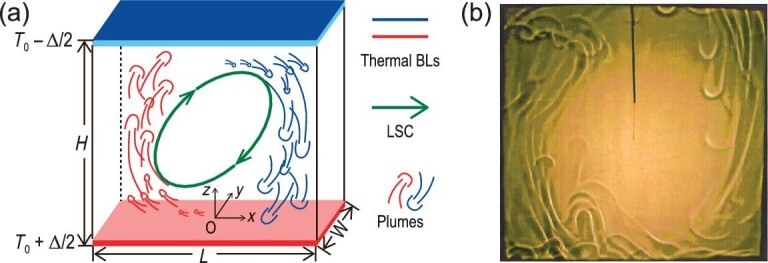
(a) Schematic drawing of turbulent RB convection in a rectangular convection cell with its height, length and width being *H, L* and *W*, respectively. The red and blue mushroom-like structures represent the hot and cold thermal plumes in the system. See the text for a detailed description. (b) Shadowgraph visualization of the spatial distribution of thermal plumes in a turbulent RB convection cell, which is taken from Shang *et al.* [28]. The measurement was made in a cylindrical convection cell at *Ra* = 6.8 × 10^8^ and Γ = 1 with dipropylene glycol (*Pr* = 596) being the working fluid.

From the classical point of view, transport efficiency of wall-bounded turbulent flows is primarily limited by a thin fluid layer adjacent to the boundaries of the systems, i.e. the boundary layer (BL; see Fig. 1 below for an illustration). The flow inside the BL is laminar (albeit fluctuating) and thus dominated by molecular diffusion process, which bottlenecks the transport capacity of relevant quantities, such as momentum and heat. Therefore, traditional methods to promote global transport in turbulent flows usually center on how to disrupt the laminar BL and make it more turbulent. This canonical approach is also prevalent in the community of thermal turbulence. As efficient heat transport is highly desirable in many industrial applications, great efforts have been devoted to finding strategies for breaking through the BL’s limitation [3].

One most widely used method is to modify the boundaries with rough elements [4–9], which can in general induce stronger interaction between the BL and the turbulent flow outside. Consequently, the BL becomes thinner, and more efficient heat transport is achieved, with the enhancement reaching as large as ∼600% [5]. However, when the height of the rough elements are small compared to the BL thickness, the hot/cold fluid may be trapped and accumulate inside the cavity regions between rough elements, resulting in heat-transport suppression [10]. This suggests that special care should be taken when using rough boundaries in real applications. Another efficient method to perturb the BL is injecting gas bubbles at the boundaries [11,12]. The generation of vapor bubbles can also disrupt the BL efficiently [13]. These methods have become promising solutions for passive heat management.

A close look at these BL-perturbation methods reveals that they essentially lead to increased emission of thermal plumes, which are the single-most-important coherent structures in thermal turbulence (see Fig. 1 for an illustration). Thanks to the extensive studies of thermal plumes over the years, it is now well accepted that these coherent structures are the primary heat carriers and play a crucial role in turbulent heat transfer processes [14,15]. Through direct visualizations and indirect measurements, the physical and geometrical properties of thermal plumes have been investigated in detail, such as their number, vorticity, geometry and ‘heat content’ [16,17]. Questions then naturally arise: Is there a one-to-one correspondence between the properties of thermal plumes and the global heat transport efficiencies? If yes, can thermal plumes be manipulated to contain more (or less) ‘heat content’ and thus used to control the heat transport in turbulent flows?

A recent discovery [18–21] provides positive answers to the questions above. It is found that, through simple geometrical confinement in a classical thermal turbulence system, thermal plumes can be manipulated to significantly enhance heat transport efficiency, despite a great reduction in the flow strength. In this review article, we introduce in detail this new mechanism of controlling turbulent heat transport via coherent structure manipulation. After explaining its underlying mechanism, we provide additional examples to demonstrate how the framework of coherent structure manipulation can be generalized to understand heat transport behaviors in various seemingly different turbulence systems. We expect that this review will interest both the fluid turbulence and heat transfer communities, and also stimulate the development of novel passive heat-transfer systems with tunable transport efficiencies.

## MANIPULATION OF THERMAL PLUMES THROUGH GEOMETRICAL CONFINEMENT

### Rayleigh–Bénard convection

The canonical model for studying turbulent heat transport is known as Rayleigh–Bénard (RB) convection, which is a buoyancy-driven flow in a fluid layer confined between two horizontally parallel plates with heating from below and cooling from above. This system can be described by the following non-dimensional governing equations under the Oberbeck–Boussinesq approximation, in which all changes in the fluid properties due to temperature variation are negligible except for the density change in the buoyancy [22]:


(1) \begin{eqnarray*} \frac{\partial {\bf {\it u}}}{\partial t} + {\bf {\it u}}\cdot \nabla {\bf {\it u}} = -\nabla p + \sqrt{\frac{\it Pr}{\it Ra}}\nabla ^2{\bf {\it u}} + T\hat{{\bf {\it z}}} + {\bf {\it f}},\\ \end{eqnarray*}

(2) \begin{eqnarray*} \frac{\partial T}{\partial t} + {\bf {\it u}}\cdot \nabla T = \sqrt{\frac{1}{{\it Ra}{\it Pr}}}\nabla ^2T, \end{eqnarray*}

(3) \begin{eqnarray*} \nabla \cdot {\bf {\it u}} = 0. \end{eqnarray*}

The last term in Equation (1) denotes an additional body force other than the buoyancy. The Rayleigh number *Ra* = α*g*Δ*H*^3^/νκ and the Prandtl number *Pr* = ν/κ in the equations above are the two control parameters, where Δ is the temperature difference across the fluid layer; *g* is the gravitational acceleration; α, ν and κ are the isobaric thermal expansion coefficient, the kinematic viscosity and the thermal diffusivity of the working fluid at the mean temperature *T*_0_, respectively. As convection systems in most situations have finite sizes, the aspect ratio Γ (lateral size of the system over its height *H*) also comes into play as the third control parameter.

Despite its simple configuration, RB convection is rich in physics and has played a paradigmatic role in the study of a wide range of subjects, such as hydrodynamic stability [23], non-linear dynamics [24], turbulence theory [25] and convection phenomena in geo-astro-physical systems [26]. The present article focuses on the heat transport in turbulent RB convection, which is usually characterized by the Nusselt number $\it Nu$ (the ratio of the total heat flux over that transported by conduction alone). Over the past decades, a large number of studies have been carried out to address how the $\it Nu$ number depends on the above three control parameters of the system. Progress in this direction has been reviewed mostly from the classical view of wall-bounded turbulence [14,15,27]. Because of the space limitation, we only give a brief introduction here.

Figure 1 shows a schematic drawing of turbulent RB convection and a typical shadowgraphic image obtained from experiment [28]. It is seen that the thermal plumes, after ejecting from the thermal BLs, organize themselves into a circulation roll that spans the height of the convection cell, which is known as the large-scale circulation (LSC). As Γ is increased to larger values, the LSC evolves from a single-roll structure to a multi-roll pattern, but the heat transfer efficiency is found to be insensitive to the change in large-scale flow structures and dynamics [29–32]. Direct evidence for this insensitivity is provided by some novel experiments, where the LSC is strongly altered by placing some obstacles inside the system but the $\it Nu$ number changes little [33,34]. These findings are consistent with the conventional view aforementioned that the most efficient way to change turbulent heat transport is to perturb the BL directly. For a detailed discussion of heat transfer in turbulent RB convection with Γ around one and larger, see the review by Ahlers *et al.* [14].

### Confinement-induced heat-transport enhancement

**Figure 2.**
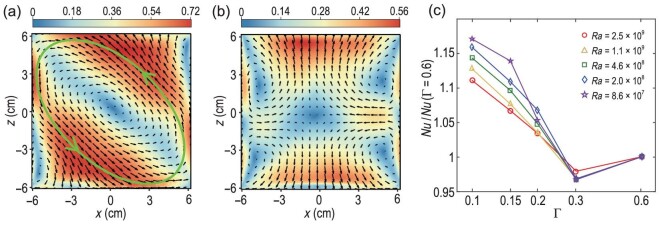
Mean velocity fields at the middle vertical plane of two rectangular RB convection cells with (a) Γ = 0.3 and (b) Γ = 0.15, adapted from Huang and Xia [20]. These velocity fields were measured by the particle image velocimetry technique [35] at *Ra* ≃ 10^9^ and *Pr* = 4.3. The velocity is coded in both color and vector length in units of cm/s. The green line with arrows in (a) is used to highlight the LSC in the convection cell. (c) Experimentally measured compensated $\it Nu$ as a function of Γ for different $\it Ra$ at *Pr* = 4.3, adapted from Huang *et al.* [18]. The solid lines in (c) are drawn to guide the eye.

While the studies of turbulent convection cells with large Γ are believed to be relevant to geo-astro-physical systems, many applications in engineering (e.g. micro-electronic cooling) take place in confined spaces with small values of Γ. However, for a long time, how turbulent flows transport heat in highly confined geometries has received little attention. Recently, there has been a number of studies using rectangular convection cells to confine the LSC such that its motion becomes quasi-two-dimensional [36–38]. In these studies, the aspect ratio of the convection cells in the *x* direction [see Fig. 1(a)] Γ_*x*_ = *L*/*H* is generally unity, whereas that in the *y* direction Γ_*y*_ = *W*/*H* (hereafter simply Γ) is in general not smaller than 0.3. Further, reducing the width and thus Γ of the convection cell to smaller values would increase the frictional drag from the lateral walls, resulting in a slower flow. This is confirmed by direct experimental measurements of the velocity field, as shown in Fig. 2(a) and (b). By narrowing the width of laterally confined Rayleigh–Bénard cells, it is found that the maximum velocity is reduced by $22\%$ when Γ decreases from 0.3 to 0.15, and the single-roll LSC disappears [18,20].

For turbulent flow with a slower motion, one would ordinarily expect that the vertical transport properties should be inhibited. Therefore, it came as a big surprise that the heat transfer efficiency increases significantly in spite of a large reduction in the flow strength [18]. As shown in Fig. 2(c), the $\it Nu$ number first decreases slightly as Γ changes from 0.6 to 0.3, and then increases unexpectedly for all the cases with Γ < 0.3. It was later found that the $\it Nu$ enhancement can be as large as $\sim\! 20\%$, while the flow strength is reduced by up to 60% [19]. Because convective heat transfer also depends on the temperature field, this counterintuitive phenomenon suggests that something must have happened to the temperature field to offset the effect of a slower flow.

Indeed, local temperature measurements in the bulk flow showed that the temperature fluctuations exhibit a significant increase with decreasing Γ, and its probability density distribution function changes from an exponential shape to a Gaussian-like one [20]. These results suggest that the properties of thermal plumes have been changed. To find out what happened to these heat-carrying objects, one needs to obtain the velocity and temperature fields simultaneously. However, this task remains inaccessible to experiments even with state-of-the-art techniques in terms of resolution and accuracy.

**Figure 3.**
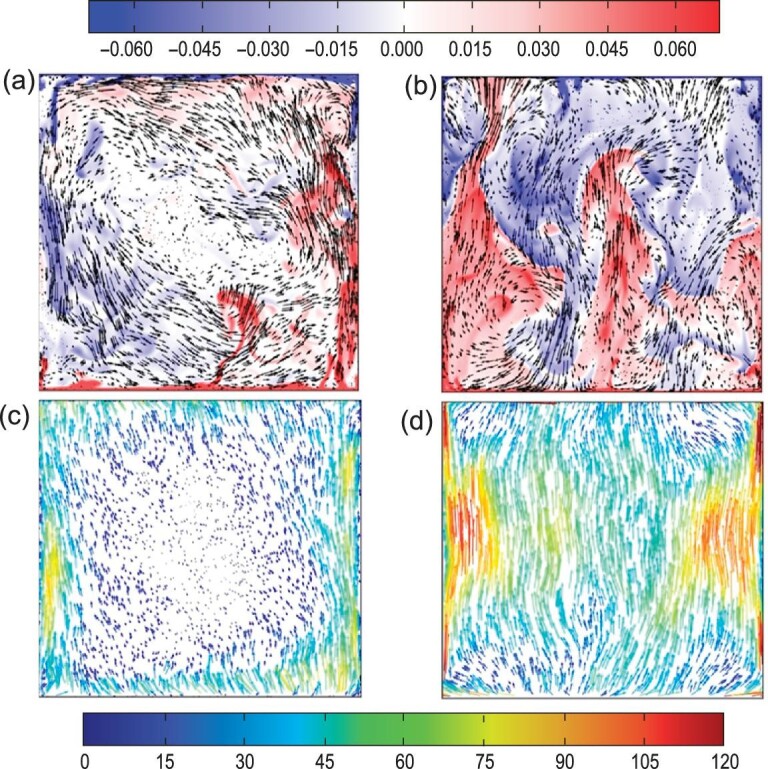
Instantaneous temperature-velocity fields (a and b) and time-averaged non-dimensional heat flux fields (c and d) at the middle vertical plane of two rectangular RB convection cells: (a and c) Γ = 1/2 and (b and d) Γ = 1/8. The results were obtained by direct numerical simulations at *Ra* = 10^9^ and *Pr* = 4.3. The temperature is color coded in units of Δ (the temperature difference across the fluid layer), and the velocity is coded in vector length and in units of the free-fall velocity $\sqrt{\alpha g \Delta H}$. The heat flux is coded in both color and vector length. Figure taken from Huang *et al.* [18].

With the rapid development of computational power and numerical methods, direct numerical simulation (DNS) has become a powerful tool for studying thermal turbulence [39]. By performing detailed DNS studies, the changes in the dynamics and morphology of thermal plumes are then clearly revealed. The red and blue structures shown in the top panel of Fig. 3 represent the hot and cold thermal plumes, respectively. In the large Γ cell, thermal plumes predominantly move along the periphery of the system; when it comes to the strongly confined case, they preferentially rise up (fall down) in random locations and pass through the bulk region in a more straightforward way. As a result, the heat transport process is no longer confined to the periphery region but takes place in the entire cell (see the bottom panel of Fig. 3). Moreover, these primary heat carriers become more coherent and energetic in the strongly confined geometry, as indicated by their more extended size. Therefore, they experience less heat loss while traversing across the convection cell and are thus able to warm up (cool down) the top (bottom) BL more effectively, leading to a net increase in the global heat transfer.

It is noteworthy that the width of the convection cell for the strongest confined case is still much larger (by at least one order of magnitude) than the BL thickness, so geometrical confinement does not perturb the BL directly. It is the changes in thermal plumes that are responsible for the heat-transport enhancement. This striking finding demonstrates for the first time how coherent structures in turbulent flow can influence global heat transport. Then the key questions arise: Why do thermal plumes become more coherent and more energetic through geometrical confinement? Can their coherency be manipulated by geometrical confinement?

### Plume condensation and universal heat transport properties

As thermal plumes are generated from the thermal BL, it would be illuminating to examine how geometrical confinement influences these ‘elementary plumes’ after their ‘birth’. Because of the top–bottom symmetry (or the cold–hot symmetry equivalently) of the RB system, we take hot plumes as an example to elucidate their evolution processes.

**Figure 4.**
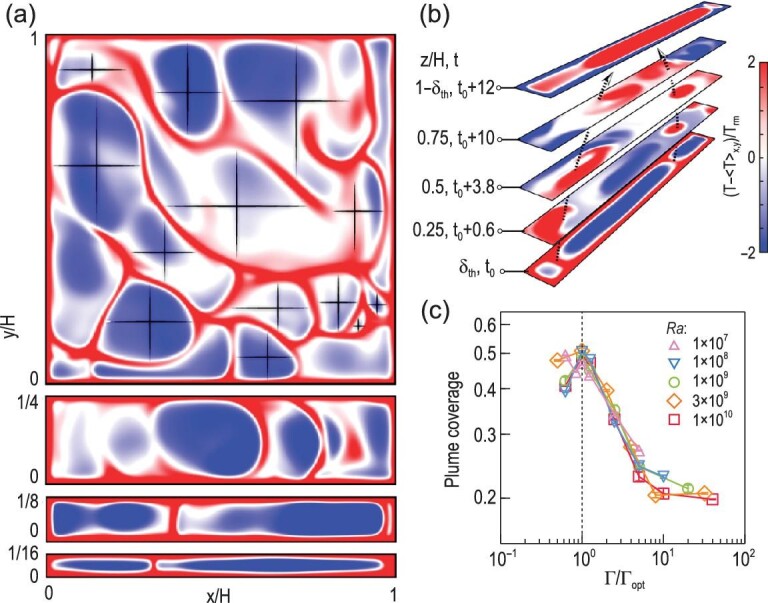
(a) Instantaneous temperature fields in a horizontal plane at the edge of the thermal BL close to the bottom boundary (*Ra* = 10^8^). From top to bottom, the aspect ratios are Γ = 1, 1/4, 1/8 and 1/16, respectively. The crosses indicate the free-moving spacing of ‘elementary plumes’ in the case without confinement effect. (b) Time sequence of temperature fields at different heights of the cell with Γ = 1/10, which is the optimal aspect ratio Γ_*opt*_ for maximum heat transport at *Ra* = 10^8^. The black dashed arrows illustrate how ‘elementary plumes’ evolve into a giant plume as they travel across the cell from certain time *t*_0_ to *t*_0_ + 12 (in free-fall time units). (c) Percentage of area occupied by thermal plumes at the edge of the thermal BL as a function of Γ/Γ_*opt*_ for different $\it Ra$. All the results were obtained by direct numerical simulations at *Pr* = 4.3. The temperature fields in (a) and (b) share the same color coding. Figure adapted from Chong *et al.* [19].

Figure 4(a) shows horizontal slices of instantaneous temperature fields at the edge of the thermal BL. It is seen that in the Γ = 1 cell the ‘elementary plumes’ [red sheet-like structures in Fig. 4(a)] exhibit cellular structures with large separations, so they can move freely in the horizontal plane before being taken away by the LSC that exists at this Γ. With the increasing degree of confinement, the width of the system becomes comparable to and even smaller than the free-moving spacing of the ‘elementary plumes’. Consequently, the horizontal motion of these ‘elementary plumes’ is forced into one dimension perpendicular to lateral walls. Therefore, they have nowhere to go after ‘birth’ but to collide with each other head-on, and merge into larger plumes before going upwards. Moreover, as illustrated in Fig. 4(b), the larger plumes further merge with likewise plumes and grow in size as they travel upwards, and finally condense into a giant plume of system size. (This process of plume condensation is better illustrated in the movies, which can be found in the supplementary materials of the original articles [18,19].)

The condensation process not only explains why thermal plumes become more coherent through geometrical confinement, but also implies that their coherency could be quantified by their size (or, equivalently, area). Figure 4(c) shows the percentage of area occupied by the coherent plumes (plume coverage) at the edge of the thermal BL. It is seen that once Γ is smaller than a certain value, which is determined by the free-moving spacing of ‘elementary plumes’ discussed above, all the data start to rise significantly and nearly collapse onto a single curve. This collapse is achieved by normalizing the aspect ratio with Γ_*opt*_, corresponding to the maximum size that ‘elementary plumes’ can condense into.

**Figure 5.**
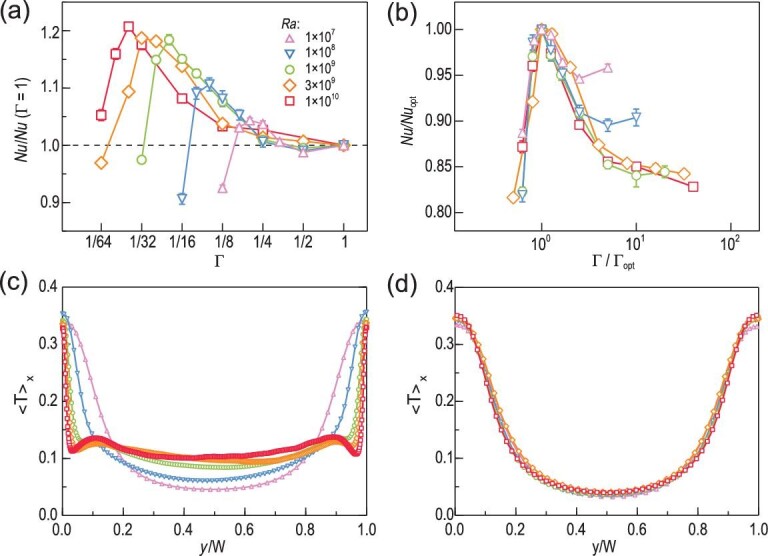
Top panels: (a) compensated $\it Nu$ versus Γ and (b) *Nu*/*Nu_opt_* versus Γ/Γ_*opt*_ for different $\it Ra$. Here, *Nu_opt_* is the optimal heat transfer efficiency occurring at Γ_*opt*_. Bottom panels: mean temperature profiles along the lateral direction (*y* direction) at the edge of the bottom thermal BL for different *Ra*: (c) Γ = 1/4; (d) Γ = Γ_*opt*_. The results were obtained by direct numerical simulations at *Pr* = 4.3. The symbols for different $\it Ra$ are indicated in (a) and the lines are drawn to guide the eye. Figure adapted from Chong *et al.* [19].

Interestingly, if the non-trivial *Nu*-Γ dependence curves [Fig. 5(a)] are re-scaled with Γ_*opt*_ and the corresponding heat transfer efficiency *Nu_opt_*, one can obtain an almost collapse of heat transport properties [Fig. 5(b)]. These universal behaviors confirm that the coherency of thermal plumes can indeed be quantified by their size, which is further connected to global heat transport. Specifically, the increased size in thermal plumes leads to the enhancement in heat transfer, and the maximum heat transfer efficiency is achieved when the plume size reaches maximum.

The universality can be found in the thermal properties at the BL as well. In contrast to the cases at fixed Γ, where the temperature profiles at the BL differ drastically for different $\it Ra$ [Fig. 5(c)], those profiles at Γ = Γ_*opt*_ fall on top of each other excellently, regardless of the $\it Ra$ numbers [Fig. 5(d)]. Moreover, the approximately flat bottom of these collapsed temperature profiles is about 0.6 of the cell width, which is the same as the averaged width of the giant plumes at Γ_*opt*_ [19]. In fact, as long as the ratio Γ/Γ_*opt*_ has the same value, both the temperature profiles and the thickness of the thermal BL exhibit universal shapes that are similar to the morphology of thermal plumes [19]. These universal behaviors signal that the system has entered a new regime that originates from the geometrical properties of thermal plumes. This regime is different from the classical regime of turbulent RB convection, where the BL properties are largely influenced by the large-scale flow. In this context, we call the new regime the plume-controlled regime.

In the plume-controlled regime, there is a one-to-one correspondence between the global heat transport and the coherency (quantified by the geometrical properties) of thermal plumes. Therefore, one can manipulate these coherent structures through simple geometrical confinement, which in turn leads to the control of global heat transport. This new paradigm of controlling turbulent transport through coherent structure manipulation is fundamentally different from the traditional approach based on direct BL perturbations. We remark that flow strength is being reduced monotonically as the degree of confinement increases. The decoupling between heat transport and momentum transport is an important characteristic of the plume-controlled regime.

### Phase diagram and implications for industrial applications

**Figure 6.**
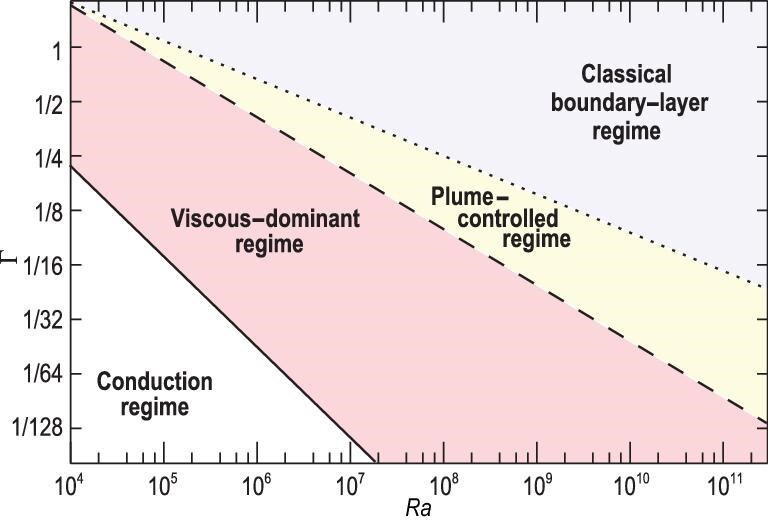
Phase diagram of different heat transfer regimes in a rectangular RB system at *Pr* = 4.3 (adapted from Chong and Xia [21]). The dotted line Γ_*bl*_ = 12.42*Ra*^−0.21^ is determined by the free-moving spacing between ‘elementary plumes’. The dashed line Γ_*opt*_ = 29.37*Ra*^−0.31^ is identified by the optimal aspect ratio for achieving maximum heat transport. The solid line Γ_*c*_ = 21.96*Ra*^−0.5^ indicates the onset of convection.

The discovery of the plume-controlled regime is particularly insightful for the design of passive cooling devices. For example, the fin-array configuration, which is widely used in electronic cooling devices, can also be applied to other engineering scenarios with large extended space. While this kind of configuration can increase the heat-exchange area, the added surfaces also increase the degree of spatial confinement for the thermal convection, so the fin-fin spacing is one key parameter for optimizing the heat removal ability of the fin-array configuration [40,41]. To provide a guideline for potential industrial applications, we summarize the phase diagram of different heat transfer regimes in Fig. 6.

First of all, the transition from the classical boundary-layer regime to the plume-controlled regime is determined by the free-moving spacing of ‘elementary plumes’ at the edge of the thermal BL. Once the cell width is comparable to the free-moving spacing, the ‘elementary plumes’ begin to condense and the plume-controlled regime sets in. In the plume-controlled regime, thermal plumes grow in size and become more coherent with increasing spacial confinement, which is responsible for the enhancement in global heat transport. The condensation process eventually leads to the formation of giant plumes of system size, corresponding to the optimal aspect ratio for maximum heat transport efficiency. Further confinement will lead to the viscous-dominant regime, in which the flow is dominated by viscous damping from the lateral walls and the heat transfer rate drops sharply. It is noteworthy that the width of the plume-controlled regime grows with increasing $\it Ra$, suggesting that there is more room to control heat transport via manipulating thermal plumes at larger $\it Ra$.

The phase diagram in Fig. 6 is obtained in rectangular convection cells by narrowing the width only. If the geometrical confinement is applied to both lateral directions, a recent study [42] showed that the critical $\it Ra$ number for the onset of convection follows a power law of *Ra_c_* ∼ 1708(1 + *C*/Γ^2^)^2^ under Oberbeck–Boussinesq conditions, where *C* is a constant that depends on the shape of the convection cells and the lateral temperature boundary conditions. It is not clear at this stage how the boundaries of the plume-controlled regime (dotted and dashed lines in Fig. 6) will be reshaped in convection systems with other geometries. Further studies are required to generalize the present phase diagram.

The transition between different heat transfer regimes depends on the *Pr* number as well. In contrast to the findings at *Pr* = 4.3, no significant $\it Nu$ enhancement was observed for *Pr* ≃ 0.8 [43]. Since the working fluids (and thus the *Pr* number) used in practice vary widely, it is essential to investigate the role of *Pr* in geometrically confined RB convection. A recent numerical study has explored this aspect [44]. By changing the *Pr* number from 0.1 to 40, it was found that the LSC is robust against the geometrical confinement for small *Pr* (say *Pr* < 0.5 at *Ra* = 10^8^) and thus no plume-controlled regime set in. It was further found that the optimal aspect ratio exhibits a power-law relation with *Pr* as Γ_*opt*_ = 0.11*Pr*^−0.06^, which provides a more complete picture of the effects of geometrical confinement on RB convection.

While the results obtained from the studies above can provide a guideline for the design of passive cooling devices, real engineering practices are always more complex. For example, temperature differences are seldom small enough for the Oberbeck–Boussinesq approximation to hold and the flows are often dominated by temperature-dependent fluid properties [45,46]. Furthermore, cooling devices with complex shapes, such as non-regular branched fins, are also not uncommon in practice [47,48]. Therefore, more careful and systematic tests are required in real applications.

## MANIPULATING COHERENT STRUCTURES THROUGH DYNAMICAL PROCESSES

We have shown how the coherency of thermal plumes in RB convection can be manipulated through simple geometrical confinement. The key mechanism is to restrict the free-moving spacing of thermal plumes, which results in pronounced changes in the plume morphology. In fact, the morphology of thermal plumes in RB convection cells can also be manipulated by adding physical obstacles inside the system, such as partitioned walls [49], suspended honeycombs [50], a porous media structure [51] and even a single straight barrier [52]. All these methods have achieved heat transfer enhancement accompanied by the formation of more coherent thermal plumes. Since the underlying mechanism of thermal plume manipulation is to constrain their dynamics, one can alternatively realize this purpose via dynamical processes. Below we introduce some examples in this direction.

### Thermal turbulence with additional stabilizing forces

One efficient way to dynamically constrain the plume motion is by applying additional stabilizing force to the system. In fact, it is not uncommon in nature and engineering applications that thermal turbulence occurs in the presence of an additional stabilizing mechanism. Typical examples include rotating RB convection (RRB) [53], double diffusive convection (DDC) [54] and quasi-static magneto-convection (QMC) [55–58], with their additional stabilizing body forces being the Coriolis force for RRB, negative buoyancy for DDC and the Lorentz force for QMC. The corresponding non-dimensional parameters to quantify the degree of stabilization are respectively the reciprocal Rossby number 1/$\it Ro$ (the ratio of the Coriolis force to buoyancy), the buoyancy ratio Λ (the ratio of the buoyancy force induced by the temperature gradient to that by the salinity gradient) and the Hartmann number $\it Ha$ (proportional to the strength of the applied magnetic field). It is obvious that the detailed mechanisms to constrain the plume dynamics in these systems are completely different. However, the mechanisms for transport enhancement in these seemingly different systems are found to be universal and can be understood in terms of coherent structure manipulation in a unified way [55,59].

**Figure 7.**
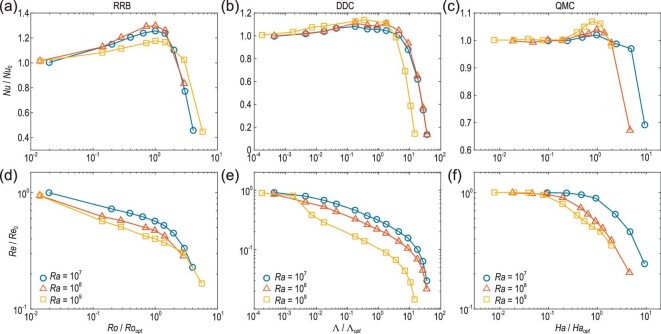
Compensated Nusselt number *Nu*/*Nu*_0_ and Reynolds number *Re*/*Re*_0_ as a function of *Ro_opt_*/*Ro* for RRB (a and d), Λ/Λ_*opt*_ for DDC (b and e) and *Ha*/*Ha_opt_* for QMC (c and f). Here, *Nu*_0_ and *Re*_0_ denote values in the cases without dynamical constraint effects; *Ro_opt_*, Λ_*opt*_ and *Ha_opt_* are the optimal parameters for achieving the maximum $\it Nu$ values with dynamical constraint effects. Both $\it Nu$ and $\it Re$ data were obtained from direct numerical simulations with similar $\it Pr$ numbers around 4–7. The data for RRB and DDC are taken from the study by Chong *et al.* [59], and those for QMC are taken from the study by Lim *et al.* [55]. These data have been re-plotted in the present figure.

Figure 7 shows how the Nusselt number $\it Nu$ and the Reynolds number $\it Re$ (characterizing the flow strength) respond to the stabilizing forces in three different systems. Note that, for the $\it Nu$ data, it refers to the heat transport in RRB and QMC, while in DDC it is the salinity transport instead. For the sake of generality, they are denoted as scalar transport here. It is seen that, for all the cases, the flow strength decreases monotonically as the stabilizing force increases, but the scalar transport first increases with increasing degree of stabilization, and then declines sharply after reaching an optimal state. By making a side-by-side comparison of these non-trivial and counterintuitive behaviors, one can immediately recognize that the behaviors of the three systems under different dynamical constraints are not only similar to each other, but also similar to the RB system under geometrical confinement. This implies that there may be a universal mechanism for understanding these distinct systems.

**Figure 8.**
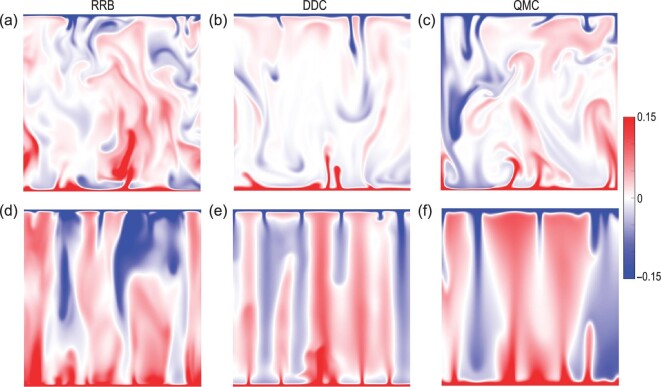
Instantaneous scalar fields at the middle vertical plane of the system: RRB (a and d), DDC (b and e) and QMC (c and f). The top and bottom panels represent the scalar fields with weak and strong dynamical constraint effects, respectively. These scalar fields were obtained from direct numerical simulations at *Ra* = 10^8^ with similar $\it Pr$ numbers around 4–7. Note that the data are taken from the studies by Chong *et al.* [59] (for RRB and DDC) and Lim *et al.* [55] (for QMC), but different snapshots are used here.

The universal mechanism emerges clearly when examining the plume morphology, as shown in Fig. 8. The red and blue structures in the figure represent the primary scalar carriers in the system, i.e. thermal or salinity plumes. It is seen that, compared to the cases without the dynamical constraint effect, the plumes are much more coherent as a result of additional stabilizing forces. These highly coherent structures can even extend the entire height of the system. Furthermore, it was found that the plume coherency, quantified by the plume coverage as introduced in Fig. 4(c), increases first and then drops as a result of increased stabilizing forces, which coincides with the change in global scalar transport [55,59]. All these findings are exactly what we have observed in the RB convection under geometrical confinement. These similar phenomena demonstrate that coherent structure manipulation can indeed be generalized to thermal turbulence systems under dynamical constraints. In other words, these seemingly different systems can be categorized to the same class of turbulent flows. In this class of turbulence, one can manipulate the coherent structures to control turbulent transport, regardless of the detailed manipulating mechanisms.

### Thermal turbulence with additional destabilizing forces

In the above examples, we have seen how stabilizing forces can be used to manipulate coherent structures in thermal turbulence. Coherent structure manipulation can also be achieved by applying additional destabilizing forces. In contrast to the stabilizing forces, which have to act in conjunction with the buoyancy to sustain the turbulent flows, the destabilizing forces can induce flow instability and even drive the turbulent flows alone. Because all the energy injected into the system must be eventually dissipated into heat, so the global balance between energy dissipation and heat transfer will be modified when additional destabilizing forces are present. This modification could result in pronounced changes in flow morphology and heat transport behaviors.

For example, applying wall shear to RB convection using the plane Couette setup [60,61] or a rotating lid [62] can lead to the formation of meandering long streaks or tornado-like coherent structures, which are accompanied by an increase in the global heat transfer. A recent numerical study found that applying horizontal vibration to turbulent RB convection can trigger more frequent emissions of thermal plumes, and achieve massive heat-transport enhancement [63,64]. It is worth mentioning that the centrifugal force in strongly rotating RB convection can also be viewed as an additional destabilizing force. By separating the hot and cold coherent structures in the system, this kind of destabilizing force can also increase the coherency of the flow and lead to a heat transfer enhancement [65,66].

**Figure 9.**
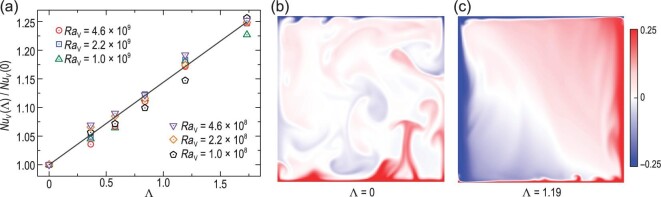
(a) Normalized vertical Nusselt number as a function of the buoyancy ratio. The solid line shows a linear fitting with *Nu_V_*(Λ)/*Nu_V_*(0) = 1 + 0.14Λ. (b and c) Snapshots of the temperature fields in the central vertical plane of a cylindrical convection cell with unity aspect ratio at two different buoyancy ratios. The results are obtained by direct numerical simulations, where the vertical Rayleigh number is fixed at *Ra_V_* = 10^8^ and the Prandtl number is *Pr* = 4.38. Figure adapted from Zhang *et al.* [70].

Another important example that has received great interest recently is the tilted Rayleigh–Bénard (TRB) convection [67–69]. In TRB convection, it is the effective horizontal buoyancy (with respect to the conducting boundaries) that serves as the additional destabilizing force. The strength of the effective horizontal buoyancy can be quantified by the horizontal Rayleigh number *Ra_H_*, or the buoyancy ratio Λ = *Ra_H_*/*Ra_V_*, where the vertical Rayleigh number *Ra_V_* is just the usual Rayleigh number describing the vertical thermal driving strength. Consequently, the Nusselt number in TRB convection can in general be expressed as a vector, with its vertical component *Nu_V_* being equivalent to the usual Nusselt number [70]. Figure 9(a) shows the normalized vertical Nusselt number *Nu_V_*(Λ)/*Nu*(0) as a function of the buoyancy ratio Λ for various vertical Rayleigh numbers *Ra_V_*. It is seen that, for fixed *Ra_V_*, the vertical heat transfer shows a monotonic increase with the buoyancy ratio. An inspection of the temperature field reveals that the effective horizontal buoyancy increases the coherency of the flow, which promotes the vertical heat transfer [Fig. 9(b) and (c)]. By combining geometrical confinement and tilting effects, a subsequent study [71] further demonstrated that promoting plume coherency is more efficient in enhancing heat transport than increasing the shear rate near the boundary, the latter of which is an often-used BL-perturbation method.

### Thermal turbulence with polymer additives

Besides additional destabilizing forces, the global balance between energy dissipation and heat transfer could be modified by energy-carrying additives in turbulent flows such as polymers. It has been known for a long time that adding a small amount of long-chain polymers into turbulent flows can lead to a dramatic reduction in viscous drag. This phenomenon occurs when the polymer relaxation time τ_*p*_ is comparable to or longer than the smallest time scale τ_η_ in turbulent flows, such that the polymers can be stretched by the turbulent flows efficiently and consequently modify the energy cascade process. This implies that the polymer-turbulence interaction is negligible when the Weissenberg number *Wi* = τ_*p*_/τ_η_ is much smaller than one. (See the review by Benzi and Ching [72] for a detailed discussion on this subject.)

**Figure 10.**
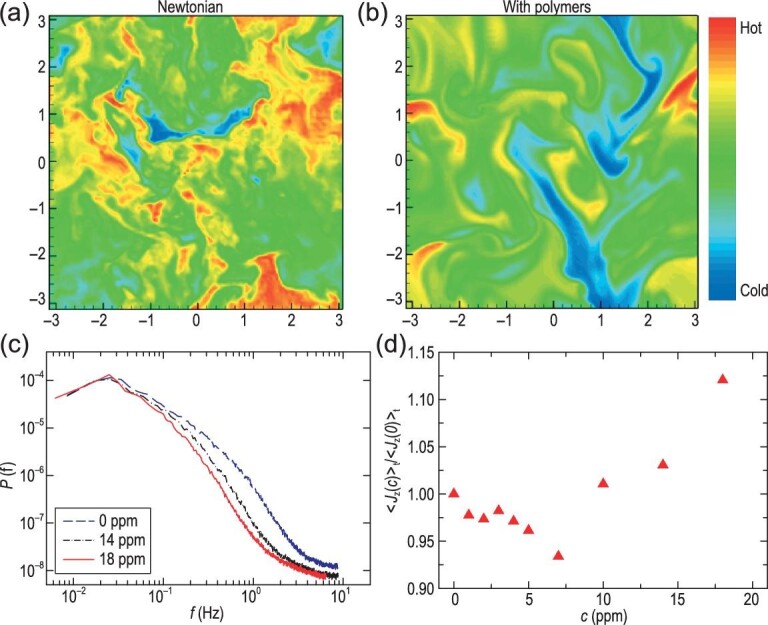
Top panels: snapshots of temperature fields obtained from direct numerical simulations of homogeneous turbulent RB convection (taken from Benzi *et al.* [75]): (a) without polymers; (b) with polymers. Bottom panels: experimental data measured in the center of a rough convection cell with *Ra* = 6.2 × 10^9^ and *Pr* = 4.3 (taken from Xie *et al.* [78]): (c) power spectral of the velocity at three different polymer concentrations, where ppm stands for parts per million by weight; (d) time-averaged vertical heat flux (normalized by the value in the Newtonian case) as a function of the polymer concentration.

For turbulent convection, the $\it Wi$ number is in general not sufficiently large to trigger the polymer-turbulence interaction globally. However, a number of recent studies revealed that even minute quantities of polymers can modify the heat transport in turbulent convection non-trivially. On the one hand, some experimental studies using RB convection cells with smooth boundaries found that polymer additives can reduce the heat transfer efficiency of the system [73,74]. The amount of heat-transfer reduction first increases with the polymer concentration, and then levels off when the polymer concentration is larger than a certain value. On the other hand, if the experiments were conducted in convection cells with rough boundaries, the global heat transport first decreases slightly and then starts to enhance progressively [74]. Heat-transport enhancement induced by polymer additives was also found in the study of homogeneous turbulent convection using direct numerical simulations, which was attributed to the increased coherency in the temperature field (see the top panel of Fig. 10) [75]. Similar phenomena, including both heat-transport enhancement and increased temperature coherency, were observed in a numerical study of Rayleigh–Taylor turbulence with polymer additives [76]. (Rayleigh–Taylor turbulence is another type of buoyancy-driven turbulent convection; see [77] for a recent review.)

The above apparently conflicting results were understood consistently with the experimental findings in a convection cell with rough boundaries [78]. Through simultaneous measurements of the local velocity and temperature, it is found that the kinetic energy of the turbulent flow is suppressed by polymers [Fig. 10(c)], but the heat transport first decreases a little and then starts to increase above a certain polymer concentration [Fig. 10(d)]. It is further revealed that the enhancement occurs concurrently with the increased coherency of thermal plumes. Meanwhile, the number of thermal plumes decreases with the polymer concentration. Based on these results, it is clear that the effects of polymer additives on turbulent convection are two-fold: firstly, they stabilize the BLs, resulting in decreased emission rate of thermal plumes and thus the heat transport [73,74,79]; secondly, they enhance the plume coherency that contributes to enhanced heat transport [75,76,78]. The two combined effects determine whether the heat transport will be enhanced or reduced.

For the two numerical simulations mentioned above [75,76], the turbulence systems used are free of BLs, in contrast to the experiments where solid boundaries are inevitable. Thus, only the second effect is present in those numerical simulations, so they can only observe heat transport enhancement accompanied by increased plume coherency. This physical picture is fundamentally the same as the framework of coherent structure manipulation. Under this light, turbulent convection with polymer additives is another example of this universal framework.

## CONCLUSIONS AND PERSPECTIVES

To summarize, we have demonstrated how the coherent structures in thermal turbulence can be manipulated through various approaches, including geometrical confinement, additional stabilizing or destabilizing forces and polymer additives. Despite their very different natures, these manipulation approaches can similarly lead to the formation of more coherent flow structures, resulting in higher efficiency of heat (scalar) transport. These universal phenomena establish a new paradigm on how coherent structure manipulation can control the transport properties in turbulent flows. As coherent structures are ubiquitous in turbulent flows, the discovery of this new paradigm is expected to inspire more studies from both fundamental research and industrial applications. In the following, we outline several directions that deserve future explorations.

First of all, the most straightforward way is to investigate this subject in a wider parameter space (e.g. pushing $\it Ra$ and $\it Pr$ numbers to higher values) and in various situations (e.g. involving more complex geometries and different types of stabilizing/destabilizing forces). It is particularly worthwhile to combine different approaches of coherent structure manipulation with traditional methods, such as combining rough boundaries with rotation [80] or geometrical confinement [81]. These more comprehensive approaches will provide certain demonstrations for engineering applications. However, any attempt in this direction would be more meaningful if it is based on realistic settings.

From the theoretical viewpoint, the underlying mechanism of coherent structure manipulation requires more in-depth investigation. The present understanding is largely based on phenomenological description. Whether, and if so how, these (and other even more) distinct systems can be unified in a theoretical framework? The answer to this question may lie in the turbulent small-scale process, which tells how the energy is balanced and transferred in turbulent flows. An intriguing turbulent small-scale process is the so-called inverse energy cascade, a phenomena that energy is transferred from small scales to large scales, which always leads to the condensation of coherent structures [82]. As inverse energy cascade has been observed in both geometrically confined and rotating thermal turbulence systems [83,84], it is plausible to develop a unified theoretical model starting from this premise.

A unified theoretical work would also help the generalization of the framework of coherent structure manipulation in other types of turbulent flows. In the present case, we consider heat transfer in thermal turbulence, but in principle it can be momentum and kinetic energy transfers in pipe flow or Taylor–Couette flow, etc. Some recent works have made attempts in this direction [85,86]. If the framework of coherent structure manipulation can be realized in other turbulence systems, it will have a far-reaching effect in both fundamental studies and applications of fluid turbulence.

Finally, we would like to discuss another important direction that is closely related to the present subject. As we mentioned at the beginning, the elementary plumes in turbulent RB convection typically organize themselves into circulatory roll(s) in large Γ cells. Some recent studies have shown that these large-scale coherent structures give rise to multiple states. Briefly, the large-scale flow can take different sizes/numbers of rolls, which give rise to different transport properties for an identical set of control parameters and the same boundary conditions of the system [87–89]. Similar phenomena have also been found in other model systems for turbulent flows such as Taylor–Couette turbulence [90], double-diffusive convection [91,92], von Karman flow [93] and Couette flow with span-wise rotation [94]. In some model studies of continent-mantle coupling of the Earth, it has been found that changing the thermal boundary conditions can lead to different organizations of large-scale coherent structures [95–97]. Moreover, if the boundaries are able to move freely, much richer flow states can be observed, which correspond to different heat transport properties [98–100]. As the coexistence of multiple turbulent states is of both fundamental interest and importance in geophysics, its underlying mechanism calls for further investigation.
